# Genome Sequence of *Streptomyces olindensis* DAUFPE 5622, Producer of the Antitumoral Anthracycline Cosmomycin D

**DOI:** 10.1128/genomeA.00541-14

**Published:** 2014-06-26

**Authors:** Juan D. Rojas, Antonio Starcevic, Damir Baranas̆ić, Maria A. Ferreira-Torres, Camilo A. Contreras, Leandro M. Garrido, Welington L. Araújo, Robson F. de Souza, Jurica Zucko, Daslav Hranueli, Paul F. Long, John Cullum, Gabriel Padilla

**Affiliations:** a Institute of Biomedical Sciences, University of São Paulo, São Paulo, Brazil; b Faculty of Food Technology and Biotechnology, University of Zagreb, Zagreb, Croatia; c Institute of Pharmaceutical Science, King’s College London, London, United Kingdom; d Department of Chemistry, King’s College London, London, United Kingdom; e Faculdade de Ciências Farmacêuticas, Universidade de São Paulo, São Paulo, Brazil; f Department of Genetics, University of Kaiserslautern, Kaiserslautern, Germany

## Abstract

*Streptomyces olindensis* DAUFPE 5622, which was isolated from a Brazilian soil sample, produces the antitumor anthracycline cosmomycin D. The genome sequence is 9.4 Mb in length, with a G+C content of 71%. Thirty-four putative secondary metabolite biosynthetic gene clusters were identified, including the cosmomycin D cluster.

## GENOME ANNOUNCEMENT

*Streptomyces olindensis* DAUFPE 5622 was isolated in the 1960s from a Brazilian soil sample. It produces the anthracycline cosmomycin D, which has antitumor activity and has attracted interest because of its distinctive glycosylation pattern (1–3). The first goal of the genome sequencing project was to determine the complete sequence of the cosmomycin biosynthetic cluster; the DNA sequence of part of the cluster was described earlier (1). A further goal was to identify additional secondary metabolite clusters in order to help isolate further interesting secondary metabolites from the strain. The genome sequences of *Streptomyces* strains often contain 20 to 30 potential secondary metabolite clusters, most of which do not correspond to known products of the strain (4). Although they have been called cryptic or silenced clusters, it is likely that they are expressed under appropriate physiological conditions. A data mining approach based on genome sequences is a promising route for isolating novel biologically active compounds.

The genome sequence was obtained using 454 pyrosequencing technology. The 650,799 reads obtained correspond to a coverage of about 29×. The assembly contains 120 contigs, with a total length of 9.4 Mb and a G+C content of 71%. The mean size of the contigs is 78 kb, and the *N*_50_ is 246 kb. The genome sequence should contain one linear plasmid of 76 kb and, if it is like other *Streptomyces* genomes, a single linear chromosome (5).

Glimmer (6) was used to determine potential protein-coding genes, which were annotated using the Rapid Annotations using Subsystems Technology (RAST) server (7). The genome was annotated using the NCBI Prokaryotic Genome Annotation Pipeline version 2.0 (8) (http://www.ncbi.nlm.nih.gov/). There were 8,287 predicted protein-coding genes and 4 rRNA gene clusters. Sixty-four tRNA sequences were predicted using the tRNAscan-SE Web server (9). Thirty-four secondary metabolite clusters were predicted using antiSMASH (10), including 7 polyketide synthases (PKS), 3 nonribosomal peptide synthetases (NRPS), 3 hybrid clusters (2 PKS-NRPS; 1 NRPS-terpene), 1 aminoglycoside, 3 siderophores, 3 bacteriocins, 3 butyrolactones, 5 terpenes, 1 ectoine, and 5 clusters of other hypothetical functions. The cosmomycin cluster is about 40 kb long and contains 42 predicted genes, whose functions will be determined by further experimental studies. The sequence of the genome of *S. olindensis* will assist in the development of genetic engineering strategies for the production of new compounds with biotechnological potential.

### Nucleotide sequence accession numbers.

This whole-genome shotgun project has been deposited at DDBJ/EMBL/GenBank under the accession no. JJOH00000000. The version described in this paper is version JJOH01000000.
